# Clinicopathologic Effects of Xenogeneic GvHD Induced by Adoptively Transferred Human-Derived T Cells in Severely Immunodeficient Mice

**DOI:** 10.34172/aim.28597

**Published:** 2024-12-01

**Authors:** Hami Ashraf, Farid Kosari, Amir Arsalan Khorsand, Samad Muhammadnejad, Vahid Mansouri, Ahad Muhammadnejad, Naser Ahmadbeigi, Seyed Mostafa Monzavi

**Affiliations:** ^1^Digestive Diseases Research Institute, Tehran University of Medical Sciences, Tehran, Iran; ^2^Chronic Respiratory Diseases Research Center, National Research Institute of Tuberculosis and Lung Diseases (NRITLD), Shahid Beheshti University of Medical Sciences, Tehran, Iran; ^3^Department of Pathology, Shariati Hospital, School of Medicine, Tehran University of Medical Sciences, Tehran, Iran; ^4^Gene Therapy Research Center, Digestive Diseases Research Institute, Tehran University of Medical Sciences, Tehran, Iran; ^5^INSERM U981, Institut Gustave Roussy, Villejuif, France; ^6^Cancer Biology Research Center, Cancer Institute of Iran, Tehran University of Medical Sciences, Tehran, Iran

**Keywords:** Adoptive immunotherapy, Evaluation, Graft, Host disease, Immunodeficient mice, Preclinical drug, Xenotransplantation

## Abstract

**Background:**

Xenogeneic graft-versus-host disease (xGvHD) is an inevitable confounder of preclinical evaluation of adoptive immunotherapies on tumor-bearing immunodeficient mouse models. This study was designed to appraise the clinical and histopathological effects caused by xGvHD in severely immunodeficient mice considering the T cell dosage.

**Methods:**

Fifty NOG mice underwent intraperitoneal injection of three different doses of human-derived total T cells, a high dose of CD8^+^T cells, or vehicle (as control). Clinical and histopathological status of the study subjects were evaluated and compared according to scoring systems.

**Results:**

In mice receiving higher doses of total T cells, the clinical severity of xGvHD was greater. However, recipients of CD8^+^T cells developed none to mild xGvHD manifestations. Higher doses of T cells were associated with poorer outcomes including premature death and more severe histopathologic damages. Greater CD3^+^T cell tissue engraftment (immunohistochemical CD3 positivity) was associated with more severe xGvHD-induced histopathological damages. Clinical xGvHD scores were significantly correlated with histopathological xGvHD scores in total and in each tissue. Mice with severe cutaneous symptoms had higher scores of xGvHD-induced histopathologic changes in the skin. Lethargy was associated with higher histopathological scores in the lungs, liver and spleen.

**Conclusion:**

In preclinical evaluations, lower doses of T cell-based therapies are associated with milder xGvHD. Development of xGvHD may be averted by the use of CD4^+^T cell-depleted grafts. Histopathological and clinical scoring systems for evaluating xGvHD are significantly correlated. The lungs and liver are reliable organs for histopathological assessment and scoring of xGvHD.

## Introduction

 Over the past few decades, immunotherapy has revolutionized cancer treatment. Adoptive transfer of *ex vivo* expanded T cells, especially in the form of antigen-specific T cells, has emerged as a robust therapeutic approach to fight against difficult-to-treat malignancies.^1,2^ Antigen-specific T cell therapies serve as anti-cancer tools with targeted graft versus tumor/leukemia effects. Among them, chimeric antigen receptor (CAR) T cell therapy has been revolutionary, as it has been shown to be highly effective in rendering refractory patients disease-free and producing durable clinical response.^3-5^ In research and development phases, these immune cell-based products should be preclinically evaluated to delineate their efficacy and safety; which in this context, drug response assays on animal models are the mainstay.

 To develop a cancer model of human origin, immunodeficient animals are essential prerequisites. There are various strains of immunodeficient mice with different levels of immunodeficiency. In this context, the most commonly used strains include nude, which lacks only T cells, and Severe Combined Immunodeficient (SCID), which lacks B and T cells. The hybrid strain of non-obese diabetic (NOD) and SCID lacks T and B cells and have impaired natural killer (NK) cells. Strains with NOD-SCID background but additionally knocked out (total or truncated) for IL-2 receptor γ chain, also known as “NOD-SCID-gamma knockout” abbreviated and marketed as NSG or NOG brand names, are characterized by lack of T, B and NK cells, reduced function of macrophages and dendritic cells and lack of complement activity.^6^ With a high degree of immunodeficiency, NSG/NOG mice are appropriate recipients of human-originated xenografts and hematopoietic cell lineages.^6-8^ They are, however, vulnerable to developing xenogeneic graft-versus-host disease (xGvHD), if undergoing adoptive transfer of immune cells. In preclinical studies using investigational adoptive immunotherapies, xGvHD is an unwanted disorder affecting the animal models and a major confounder of the study outcomes.^9,10^ When, for example, evaluating a CAR T product in preclinical phase is in focus, the inevitable development of xGvHD in the cancer model distorts the efficacy and safety results and makes the analyses unreliable.

 Both clinical and histopathological features of xGvHD have been separately studied in a number of experimental studies.^11-13^ It is still unclear how the clinical and histopathological parameters of this disorder are associated with each other and what the impacts of cell dose on each parameter are. In this study, we aimed to appraise the severity of histopathologic changes caused by xGvHD in severely immunodeficient mice and to assess their correlation with concurrent clinical manifestations concerning the dosage of T cells administered.

## Materials and Methods

###  Animals 

 This study was carried out on 8 to 12-week-old NOG mice (provided from the PDX & Preclinical Modeling Core, Digestive Diseases Research Institute, Tehran University of Medical Sciences, Iran). The mice were housed in individually ventilated cage system maintained in a strictly controlled barrier facility with 12 h light/dark cycles, 21-23 °C temperature, and 55% ± 10 humidity, and had *ad libitum* access to sterile water and dry food.

###  Intervention and Grouping

 The T cells used to treat animal subjects for the development of xGvHD models were expanded according to a protocol optimized for CAR T cell generation.^14-16^ Briefly, peripheral blood mononuclear cells (PBMCs) were collected via density-gradient centrifugation using Ficoll-Paque-Plus (GE Healthcare, UK) from a single healthy donor. Then, the PBMCs were washed with phosphate-buffered saline (PBS) twice at 200 g for 10-15 minutes to eliminate platelets. A CD8^+^T cell-enriched fraction was isolated through immunomagnetic separation by negative selection using the CD8^+^T cell isolation kit (Miltenyi Biotech, Bergisch Gladbach, Germany) according to the manufacturer’s instructions. To expand T cells (total and CD8^+^ enriched fraction), 1.6 × 10^6^ PBMCs were plated and stimulated at a 2:1 ratio with 0.8 × 10^6^ anti-CD2/CD3/CD28-coated MACSiBead^TM^ particles (Miltenyi Biotec, Germany) in a 24-well plate filled with in 2 mL PRMI medium (Bioidea, Iran) in each well containing 10% heat-inactivated FBS (Gibco, USA), 100 U/mL penicillin/streptomycin (Bioidea, Iran) and 20 IU/mL recombinant human IL-2 (Miltenyi Biotec, Germany). Half of the cell culture medium was replenished based on a three-day schedule for 15 days. In addition, restimulation of T cells with the mentioned microbeads was performed after every three rounds of medium exchange, while keeping bead-to-cell ratio constant. Half of the propagating T cells were passaged to a new well, whenever the cell density reached about 3.2 × 10^6^ cells per well. The T cell products (total T cells and CD8^+^T cell-enriched fraction) for injection contained cells resuspended in 200 μL PBS (as vehicle) and were tested to be mycoplasma free.

 Fifty mice were divided into five groups of 10 based on the intervention. Interventions included intraperitoneal injection of three different doses of total T cells (1, 5, 25 × 10^6^ cells), a dose of 25 × 10^6^ CD8^+^T cells and PBS (placebo control). The immunophenotypes of the injectable cell products were analyzed on the day of injection (15 days post-isolation). For this purpose, T cells were re-suspended in 100 μL PBS and incubated with the recommended amount of anti-human CD3-PerCP (Miltenyi Biotec, Germany), anti-human CD4-FITC/CD8-PE (Cytognos SL, Salamanca, Spain) (Figure S1, Supplementary file 1). Subsequently, the cells were processed using BD FACSCalibur^TM^ and the raw data were analyzed with the FlowJo software (FlowJo LLC, USA). Accordingly, the total T cell injectable product consisted of 94.9% CD3^+^ cells, 14.3% CD3^+^ CD4^+^ cells, and 69.5% CD3^+^ CD8^+^ cells, while CD8^+^T cell-enriched product consisted of 96.9% CD3^+^ cells, ~ 0% CD3^+^ CD4^+^ cells, and 87% CD3^+^ CD8^+^ cells.

###  Necropsy, Tissue Collection and Processing

 The animals were clinically examined on a regular basis during the maximum length of observation of 60 days. The animals were euthanized 60 days after intervention, otherwise if they reached humane endpoints. Premature euthanasia was considered for mice reaching humane endpoints before the day 60^th^ post-intervention. Humane endpoints included emaciated state based on body condition scoring, lethargy, hunched posture, any condition majorly interfering with daily activities (i.e., eating, drinking, ambulation, or elimination).^6,17^ The mice were euthanized via intraperitoneal injection of 100 mg/kg ketamine and 5 mg/kg acepromazine (both from Alfasan Co., The Netherlands) followed by cervical dislocation. After euthanasia, necropsy and gross histological analysis of the major organs and tissues (including the lungs, spleen, liver, skin and duodenum) were performed. Tissue specimens were immersion-fixed in 10% neutral buffered formalin (Sigma-Aldrich Chemie GmbH, Munich, Germany), subsequently processed for paraffin embedding, sectioned at 3 μm and stained with hematoxylin and eosin (H&E; both from Sigma-Aldrich Chemie GmbH, Munich, Germany). Microarray sections at 2 μm were obtained from tissue blocks and then immunostained with leporine anti-human CD3 (clone EP41; Máster Diagnóstica, Granada, Spain).

###  Clinical and Histopathological Scoring

 The lab operator, who clinically examined the mice and measured their weight and tumor size, was blinded to the grouping. Using a recommended scoring system,^9^ clinical severity of xGvHD was assessed on the euthanasia day. The histopathologic assessment was performed according to a recommended scoring system (Table 1), by two pathologists who were blinded to the grouping. By evaluating immunostained slides, CD3 positivity of different tissues was gauged according to Allred scoring system.^18^

**Table 1:** Scoring System for Histopathological Evaluation of Xenogeneic Graft-Versus-Host Disease

**Score**	**Liver and lung**	**Spleen**	**Intestine**	**Skin**
0.5	Minimal perivascular cuffing	Occasional necrotic cells	Occasional necrotic crypt cell, minimal infiltration in lamina propria and submucosa	Focal to diffuse basal cell layer hyperplasia (increase of the basal cell layer to more than one cell in thickness).
1	Perivascular cuffing, 1 to 2 cells in thickness, involving ≤ 15% of vessels	Necrotic/apoptotic cells, ≤ 10 cells/mm^2^ of tissue	Necrotic cells in ≤ 15% of crypts, minor infiltration of ≤ 20% of lamina propria (1 to 2 cell thickness in intermucosal areas and submucosa)	Basal cell layer vacuolization
1.5	Perivascular cuffing, 1 to 2 cells in thickness, involving ≤ 15% of vessels and infiltration into parenchyma proper	Necrotic/apoptotic cells, ≤ 10 cells/mm^2^ of tissue and occasional hemolysis	Necrotic cells in ≤ 15% of crypts, minor infiltration ≤ one third of the lamina propria (1 to 2 cell thickness in intermucosal areas and submucosa)	Epidermal and follicular dyskeratosis (nuclear pyknosis or fragmentation and cytoplasmic hypereosinophilia), epidermal lymphocytic infiltrate
2	Perivascular cuffing, 2 to 3 cells in thickness, involving 16-25% of vessels and infiltration into parenchyma proper	Necrotic/apoptotic cells, 11-20 cells/mm^2^ of tissue, and occasional hemolysis with abnormal architecture	Necrotic cells in 16-25% of crypts, infiltration of ≤ one third of the lamina propria (3 cell thickness in intermucosal areas and submucosa)	Epidermal maturation disarray; i.e., disorderly change in axis from vertical (basal) to horizontal (stratum corneum)
2.5	Perivascular cuffing, 2 to 3 cells in thickness, involving 26-50% of vessels and infiltration into parenchyma proper	Necrotic/apoptotic cells, 11-20 cells/mm^2^ of tissue, and hemolysis in ≤ 25% of the tissue with abnormal architecture	Necrotic cells in 26-50% of crypts, infiltration of less than or equal to one third of lamina propria (3 to 4 cell thickness in intermucosal areas and submucosa)	Apoptosis in epidermis/follicle, satellitosis of lymphocytes about dyskeratotic cells (peripheral aggregation of intraepidermal mononuclear cells about dyskeratotic keratinocytes)
3	Perivascular cuffing, 4 to 5 cells in thickness, involving 26-50% of vessels, peribronchiolar cuffing (2 to 3 cells) and infiltration into parenchyma proper	Necrotic/apoptotic cells, ≤ 40 cells/mm^2^ of tissue, hemolysis in 26% - 50% of tissue with abnormal architecture and areas of leukopenia involving ≤ 25% of tissue, formation of fibrous bands	Necrotic cells in > 50% of crypts, infiltration of lamina propria (5 to 6 cell thickness in intermucosal areas and submucosa) with loss of ≤ 25% of goblet cells	Dermal lymphocytic infiltrate
3.5	Perivascular cuffing, 6 to 7 cells in thickness, involving > 50% of vessels, peribronchiolar cuffing (4 to 5 cells, lung), necrotic foci (liver) and infiltration into parenchyma proper with severe disruption of structure	Necrotic/apoptotic cells, ≤ 40 cells/mm^2^ of tissue, hemolysis evident in > 50% of tissue with abnormal architecture and areas of leukopenia involving 26-50% of tissue, formation of fibrous bands	Necrotic cells > 50% of crypts, infiltration of lamina propria resulting in displacement of ≤ 50% of mucosa with loss of 50% of goblet cells	Lichen planus-like changes
4	Perivascular cuffing, ≥ 8 cells in thickness, involving greater than 50% of vessels, peribronchiolar cuffing ( > 6 cells, lung), large necrotic foci (liver), and infiltration into parenchyma proper with necrotic lesions (liver, lung);	Large areas of necrosis and hemolysis evident in > 50% of tissue with abnormal architecture and large areas of leukopenia involving > 50% of tissue	Necrotic cells in > 50% of crypts, infiltration of lamina propria resulting in displacement of > 50% of mucosa with loss of 75-100% of goblet cells	Sclerodermoid changes

###  Statistical Analysis

 Statistical analyses were performed using GraphPad Prism (GraphPad Software Inc., San Diego, USA) and IBM SPSS Statistics (SPSS Inc., Chicago, USA). Since the distributions of quantitative data were found to be normal by using D’Agostino-Pearson omnibus test, the relevant results are reported with mean and standard deviation (SD). Qualitative variables are reported as frequency and percentage. To compare quantitative data between two groups, independent samples T test, and among multiple groups, one-way ANOVA were used. Comparison of frequency counts between two groups was conducted using chi-squared test. The relationship between two quantitative data was analyzed using Pearson’s correlation coefficient. Survival analysis was performed using the Kaplan-Meier method. The log-rank (Mantel-Cox) test was applied to analyze the difference of survival curves between the groups. The level of significance was set at 0.05.

## Results

###  Clinical Findings

**Figure 1:**
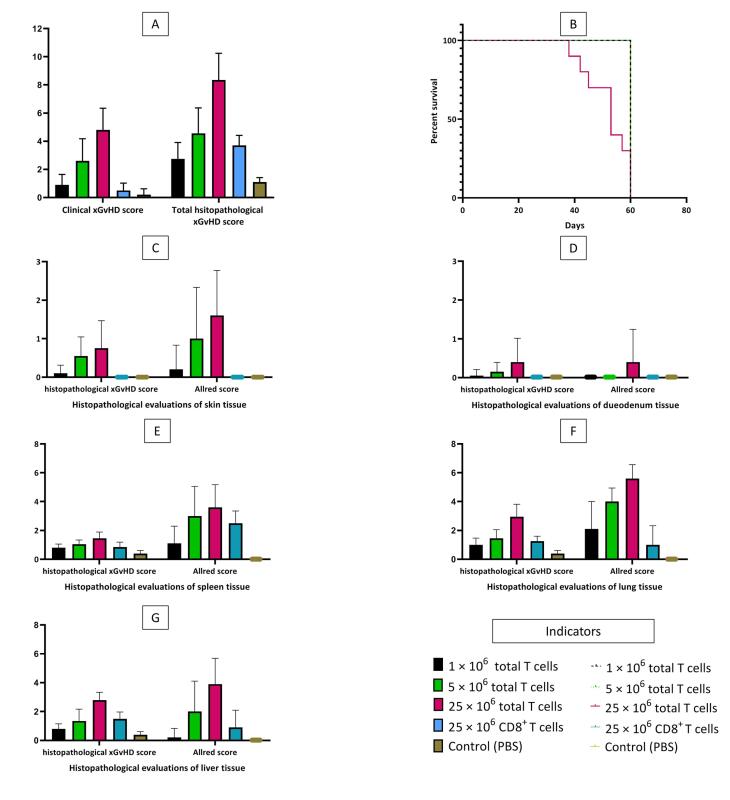
Clinical and Histopathological Xenogeneic Graft-Versus-Host Disease Scoring, and Survival Curve of the Study Subjects. **A,** mean clinical and total histopathological xGvHD scores of the study groups. **B,** overall survival of the study groups. **C,** mean histopathological xGvHD and Allred scores of the skin based on the study groups. **D,** mean histopathological xGvHD and Allred scores of the duodenum based on the study groups. **E,** mean histopathological xGvHD and Allred scores of the spleen based on the study groups. **F,** mean histopathological xGvHD and Allred scores of the lung based on the study groups. **G,** mean histopathological xGvHD and Allred scores of the liver based on the study groups.

 All T cell-treated mice (those treated with total T cells) started to develop prodromal signs of xGvHD including ruffled fur and conjunctival erythema 1-2 weeks after intervention. Control mice (PBS-treated mice) remained asymptomatic until euthanasia (few of them only developed very mild ruffled fur). In mice receiving higher doses of total T cells, the severity of xGvHD was greater. However, recipients of 25 × 10^6^ CD8^+^T cells developed none to mild xGvHD manifestations (Figure 1A). Mice receiving 25 × 10^6^ total T cells had significantly shorter lifespans (Figure 1B). All other groups survived through the maximum length of evaluation. Severe and terminal signs of xGvHD such as dermatitis (scaling or ulcerative), alopecia, severe emaciation, and lethargy (persistent recumbency) became only evident in recipients of 5 and 25 × 10^6^ total T cells.

###  Histopathological Findings

**Figure 2:**
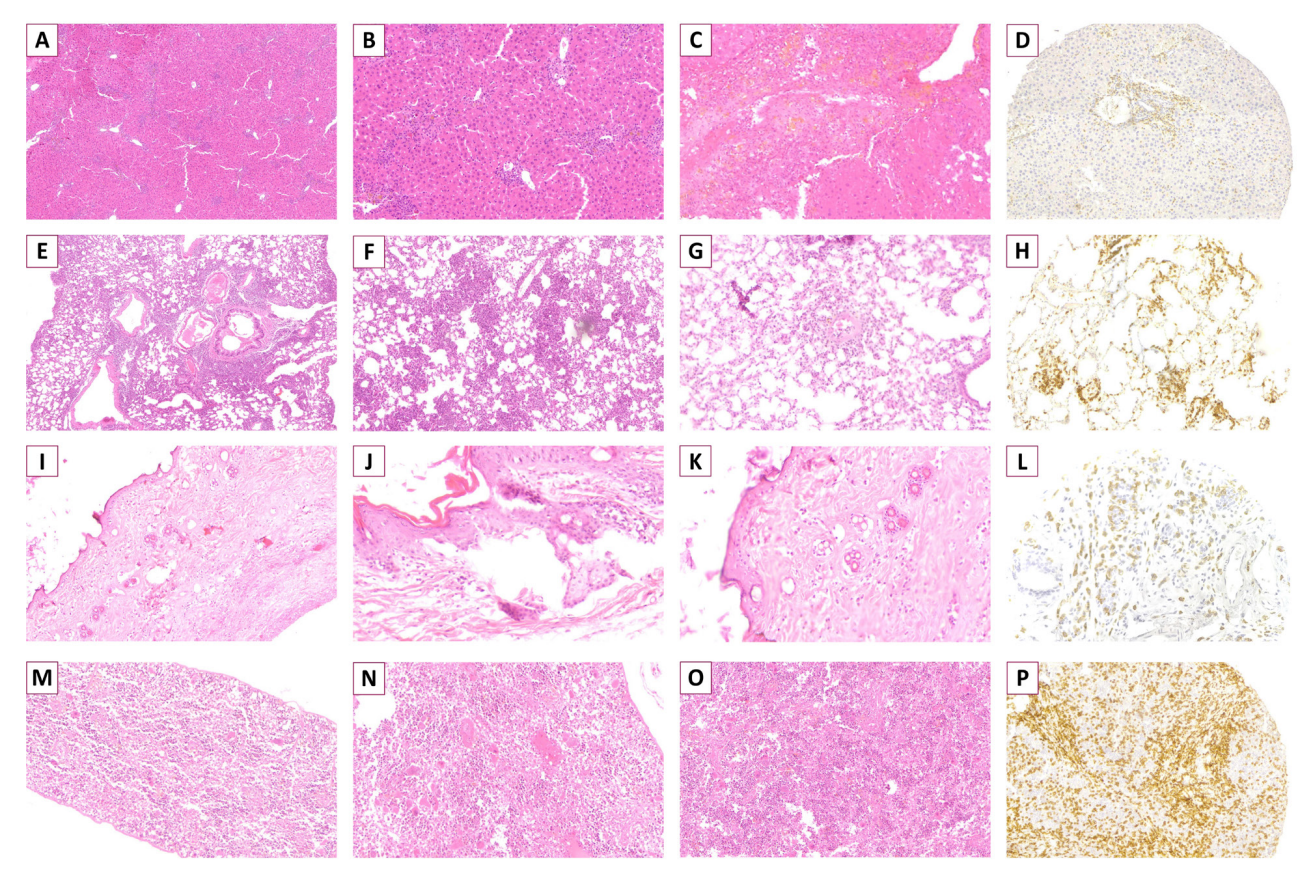
Histopathology of Xenogeneic Graft-Versus-Host Disease in Representative Tissue Specimens of Experimental Mice. **A-C, **liver specimens showing perivascular cuffing infiltrated into adjacent parenchyma proper (A) with necrotic lesions (B) and disruption of structure (C) (A-C: H&E, x400). **D,** liver specimen infiltrated by multiple human-derived CD3^+^ cells (CD3 IHC, x200). **E-G,** lung specimens showing heavy perivascular and peribronchiolar cuffing infiltrated into parenchyma proper (E) with necrotic foci (F) and disruption of structure (G) (E: H&E, x100; F and G: H&E, x400). **H,** lung specimen infiltrated by multiple human-derived CD3^+^ cells (CD3 IHC, x200). **I-K**, Skin specimen showing epidermal dyskeratosis, apoptosis in epidermis/follicle, satellitosis of lymphocytes (I: H&E, x100; J and K: H&E, x400). **L,** skin specimen infiltrated by multiple human-derived CD3^+^ cells (CD3 IHC, x200). **M-O,** spleen biopsy showing occasional apoptotic and necrotic cells with small areas of hemolysis (M: H&E, x100; N and O: H&E, x400). **P,** spleen specimen infiltrated by multiple human-derived CD3^+^ cells (CD3 IHC, x200).

 Similar to clinical findings, in mice receiving higher doses of total T cells, more severe histopathologic changes of xGvHD developed. The highest scores of xGvHD-induced histopathologic changes in the skin, duodenum, spleen, lung and liver were detected in the mice receiving 25 × 10^6^ total T cells (Figure 1C-G). According to histopathological evaluations, the majority of mice receiving 25 × 10^6^ total T cells had epidermal maturation disarray in the skin, occasional hemolysis and intermediate number of necrotic/apoptotic cells with abnormal architecture in the spleen, perivascular cuffing involving approximately half of vessels in the liver, and peribronchiolar cuffing and infiltration into parenchyma proper in the lungs (Figure 2).

**Figure 3:**
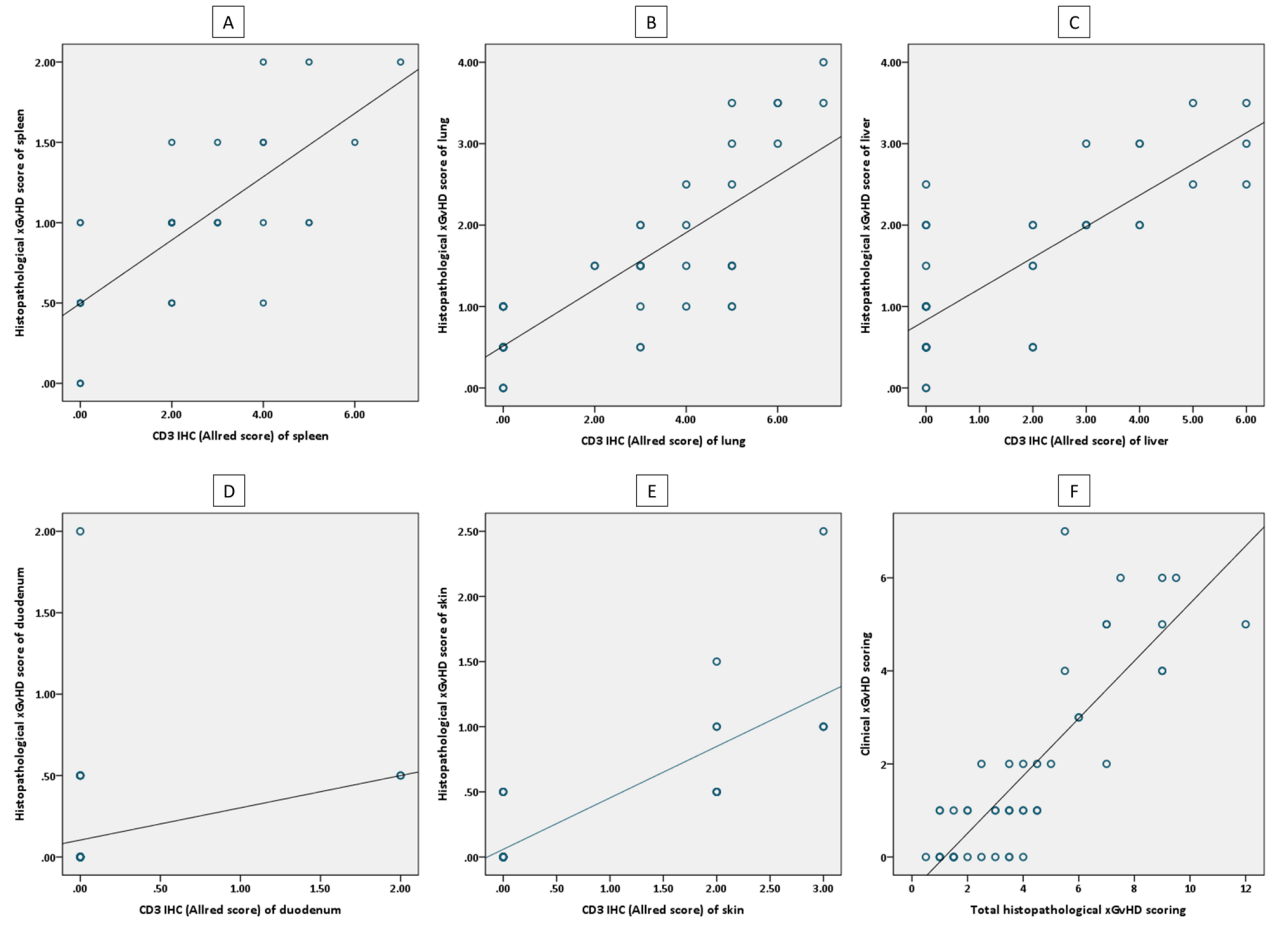
Scatterplots showing correlations between histopathological, immunohistochemical and clinical scoring. **A,** correlation of histopathological xGvHD scores and immunohistochemical Allred scores (CD3 positivity) of spleen. **B,** correlation of histopathological xGvHD scores and immunohistochemical Allred scores (CD3 positivity) of lung. **C,** correlation of histopathological xGvHD scores and immunohistochemical Allred scores (CD3 positivity) of liver. **D,** correlation of histopathological xGvHD scores and immunohistochemical Allred scores (CD3 positivity) of duodenum. **E,** correlation of histopathological xGvHD scores and immunohistochemical Allred scores (CD3 positivity) of skin. **F,** correlation of total histopathological xGvHD scores and clinical xGvHD scores.

 Interestingly, total histopathological xGvHD scores of the recipients of 1 × 10^6^ total T cells and 25 × 10^6^ CD8^+^T cells were not significantly different (Figure 1A). Greater immunohistochemical CD3 positivity (higher Allred scores) in all tissues was seen in cases with higher histopathological xGvHD scores. In other words, tissues infiltrated with higher numbers of human-derived CD3^+^T cell were affected with more severe xGvHD-induced histopathologic changes (Figure 3A-E). IHC findings revealed that the greatest extent of human-derived T cell engraftment occurred in the lungs, spleen and liver (Figure 1E-G). In all tissue specimens, the lowest rate of CD3^+^ cell engraftment was detected in mice receiving 1 × 10^6^ total T cells and 25 × 10^6^ CD8^+^T cells. Engraftment of human-derived CD3^+^ cells in the duodenum was only seen in mice receiving 25 × 10^6^ total T cells. It is of note that although very severe xGvHD-induced damages occurred in the lungs and liver (mean xGvHD score of 3-3.5) in connection with more profound infiltration of CD3^+^T cells into these tissues (mean CD3 Allred score of 5-6) (Figure 1F-G), the extent of histopathological damage in the spleen was only mild to moderate (mean xGvHD score of 1-2) despite high CD3^+^T cell engraftment (mean CD3 Allred score of 3-5) (Figure 1E). In other words, engraftment of human-derived T cells in the murine spleen did not cause severe histopathologic damages. Moreover, in mice receiving 25 × 10^6^ CD8^+^T cells with no xGvHD-induced change and CD3^+^T cell infiltration in the duodenum and skin, and limited xGvHD damage to the lungs, liver and spleen, the CD3^+^T cell engraftment was highest in the spleen.

###  Clinicopathologic Correlations

 Using correlation analysis, it was found that clinical xGvHD scoring (on the euthanasia day) is significantly correlated with total histopathological xGvHD scoring (r = 0.842, *P* < 0001) (Figure 3F). The associations of clinical xGvHD score with histopathology scores of each tissue were also significant. Nonetheless, the best correlations between histopathology scores of a single tissue and clinical xGvHD score were ascertained for the spleen, liver and lung (r > 0.8, *P* < 0001 for all).

Table 2 shows the intervention groups and outcomes plotted against histopathological xGvHD scoring in each tissue and in total and clinical xGvHD scoring. Based on the intervention, there were significant differences among histopathological scores in each tissue and in total, except for the duodenum. This means that more severe histopathologic damages occurred in the skin, lungs, spleen and liver following administration of higher T cell doses, whereas the duodenum was spared from histopathologic damages even in the recipients of the highest T cell dose. It is noteworthy that there were non-specific very minor histopathologic changes in the liver, lungs and spleen of control mice, implying that baseline minimal histopathologic changes in these organs may occur (even in untreated mice) and are negligible. Moreover, prematurely euthanized mice (i.e., some of the mice receiving 25 × 10^6^ total T cells) had significantly higher histopathological scores in the spleen, liver, skin and lung and higher clinical scores (Table 2). This implies that premature death was associated with severe histopathologic damages in the liver, lungs, spleen, and skin, along with high clinical scores (> 5).

**Table 2:** Plotting Intervention Categories and Outcomes against Histopathological and Clinical xGvHD Scoring (n = 50)

**Parameter**	**Histopathological xGvHD scoring; mean±SD**						**Clinical xGvHD scoring, mean±SD**
	**Spleen**	**Lung**	**Liver**	**Skin**	**Duodenum**	**Total**	
Intervention							
1 × 10^6^ total T cells	0.8 ± 0.3	1 ± 0.5	0.8 ± 0.3	0.1 ± 0.2	0.1 ± 0.1	2.8 ± 1.2	0.9 ± 0.7
5 × 10^6^ total T cells	1.1 ± 0.3	1.5 ± 0.6	1.4 ± 0.8	0.6 ± 0.5	0.2 ± 0.2	4.6 ± 1.8	2.6 ± 1.6
25 × 10^6^ total T cells	1.5 ± 0.4	3.0 ± 0.9	2.8 ± 0.5	0.8 ± 0.7	0.3 ± 0.4	8.4 ± 1.9	5.0 ± 1.6
25 × 10^6^ CD8^+^T cells	0.9 ± 0.3	1.3 ± 0.4	1.5 ± 0.5	0	0	3.7 ± 0.7	0.5 ± 0.5
PBS (placebo)	0.4 ± 0.2	0.4 ± 0.2	0.4 ± 0.2	0	0	1.1 ± 0.3	0.2 ± 0.4
*P* value^a^	< 0.001	< 0.001	< 0.001	< 0.001	0.056	< 0.001	< 0.001
Outcome							
Survival	0.8 ± 0.4	1.1 ± 0.7	1.1 ± 0.7	0.2 ± 0.3	0.1 ± 0.3	3.3 ± 2.0	1.2 ± 1.4
Premature euthanasia	1.5 ± 0.4	3.2 ± 0.8	3.0 ± 0.5	0.9 ± 0.8	0.1 ± 0.2	8.8 ± 2.0	5.9 ± 0.7
*P* value^b^	< 0.001	< 0.001	< 0.001	0.044	0.845	< 0.001	< 0.001

xGvHD, xenogeneic graft-versus-host disease. ^a^Comparison among intervention groups analyzed with one-way ANOVA. ^b^Comparison between outcomes analyzed with independent samples T test.

 To assess the relationship between severe xGvHD clinical manifestations and xGvHD-induced histopathologic changes in vital organs, a subset analysis was performed on the mice receiving 5 and 25 × 10^6^ total T cells. It was found that there were associations between some severe clinical signs on the euthanasia day and histopathologic scoring (Table 3). Mice with severe cutaneous symptoms (severe coat ruffling, dermatitis and alopecia) had higher scores of xGvHD-induced histopathologic changes in the skin. Lethargy (persistent recumbency) was associated with higher histopathological scores in the lungs, liver and spleen (with the greatest level of significance for the lungs) (Table 3). Paleness and severe emaciation were not significantly correlated with histopathologic changes in any of the tissue specimens. Nonetheless, mice with severe emaciation had higher histopathological scores in the liver, though the p value only approached the level of significance (*P* = 0.059).

**Table 3:** Plotting Severe Clinical Signs against Histopathological xGvHD Scoring (Subset Analysis, n = 20)

**Parameter**	**Histopathological xGvHD Scoring (Mean±SD)**					
	**Spleen**	**Lung**	**Liver**	**Skin**	**Duodenum**	**Total**
Severe ruffled fur						
Present	1.5 ± 0.4	2.6 ± 1.2	2.6 ± 0.6	1.0 ± 0.8	0.2 ± 0.3	8.0 ± 2.5
Absent	1.1 ± 0.4	2.0 ± 1.0	2.1 ± 1.0	0.4 ± 0.4	0.3 ± 0.5	5.8 ± 2.5
*P* value^*^	0.076	0.304	0.086	0.043	0.517	0.088
Dermatitis						
Present	1.6 ± 0.4	3.3 ± 0.6	2.7 ± 0.7	1.2 ± 0.8	0.1 ± 0.2	5.6 ± 2.4
Absent	1.1 ± 0.4	1.8 ± 0.9	1.8 ± 1.0	0.5 ± 0.4	0.3 ± 0.5	8.9 ± 1.9
*P* value^*^	0.066	0.004	0.109	0.015	0.352	0.013
Alopecia						
Present	1.5 ± 0.4	2.9 ± 1.1	2.6 ± 0.7	1.2 ± 0.7	0.2 ± 0.3	8.3 ± 2.2
Absent	1.1 ± 0.4	1.9 ± 0.9	1.9 ± 1.1	0.4 ± 0.4	0.3 ± 0.5	5.6 ± 2.5
*P* value^*^	0.076	0.043	0.142	0.009	0.517	0.034
Paleness						
Present	1.2 ± 0.3	2.3 ± 1.3	2.0 ± 0.0	0.8 ± 0.3	0.3 ± 0.3	6.7 ± 1.0
Absent	1.3 ± 0.4	2.2 ± 1.1	2.1 ± 1.1	0.6 ± 0.7	0.3 ± 0.5	6.4 ± 2.9
*P* value^*^	0.716	0.820	0.893	0.586	0.824	0.883
Severe emaciation						
Present	1.3 ± 0.3	2.4 ± 1.1	2.7 ± 0.7	0.8 ± 0.4	0.1 ± 0.2	7.3 ± 1.9
Absent	1.2 ± 0.5	2.1 ± 1.1	1.9 ± 1.0	0.6 ± 0.7	0.3 ± 0.5	6.2 ± 2.9
*P* value^*^	0.764	0.638	0.059	0.539	0.352	0.424
Lethargy						
Present	1.6 ± 0.4	3.5 ± 0.3	3.0 ± 0.5	1.0 ± 0.7	0.2 ± 0.3	9.3 ± 1.5
Absent	1.1 ± 0.3	1.6 ± 0.7	1.7 ± 0.9	0.7 ± 0.5	0.3 ± 0.4	5.2 ± 2.0
*P* value^*^	0.014	< 0.001	0.004	0.101	0.499	< 0.001

xGvHD, xenogeneic graft-versus-host disease. ^*^Comparison of scores between subjects having (present) and not having (absent) selected clinical signs (parameters) analyzed with independent samples T test.

## Discussion

 In this study, the severity of histopathologic changes caused by xGvHD in the vital organs of severely immunodeficient mice following adoptive transfer of human-derived T cells was appraised and their correlations with concurrent clinical findings were assessed. Moreover, the reliability of a scoring system for histopathologic changes of xGvHD in experimental mice following adoptive immunotherapy was ascertained.

 Adoptive T cell transfer, using either unmanipulated or cytokine-activated or genetically-manipulated T cells (e.g., CAR T cells), is a burgeoning strategy for treating different types of malignancies. However, before entering into the market and in research and development phases, the xGvHD owing to the impacts of human-derived T cells on host tissues can obscure the real effects of the treatment, i.e., graft-versus-tumor (GvT) effects and other safety issues.^9,19^ Moreover, xGvHD may cause increased activation and proliferation of T cells, leading to an overestimated antitumor efficacy.^20^ Furthermore, xGvHD can result in severe morbidities and premature mortality in mouse models causing lifespan shortening, and can therefore hamper evaluation of long-term effects or benefits of a T cell therapy.^21^ Hence, optimal dose selection and careful assessment of clinical and histopathological impacts of xGvHD in every preclinical adoptive immunotherapy research on immunodeficient mice seem necessary.

 In human-derived tumor-bearing mouse models, severe xGvHD reactions can lead to greater tumor clearance, because of both GvT and GvH effects. To be more precise, GvH can augment GvT effects of an adoptive immunotherapy in tumor-bearing immunodeficient mice.^10^ However, understanding and measurement of the exact cause of tumor shrinkage in a model is challenging, since differentiating GvT from GvH effects is difficult.^10,22^ This overlap can be confounding to interpret whether a novel T cell therapy is indeed efficacious against a tumor or whether the observed effects were mainly due to GvH.^23^

 According to our findings, all total T cell-treated mice showed some degrees of xGvHD regardless of the dose. Moreover, we found that higher doses of T cells were associated with more severe clinical signs and histopathological changes following xGvHD. In addition, histopathological xGvHD scores were significantly correlated with IHC Allred scores, supporting that greater engraftment of human-derived T cells had caused more severe histopathological damages. Interestingly, however, mice receiving a high dose of CD8^+^T cells did not develop severe clinical symptoms and histopathological changes. This is generally due to the fact that naïve CD4^+^T cells have a central role in induction of GvHD,^24^ especially in murine models^25,26^; and therefore, xenotransplantation of a CD4^+^T cell-depleted graft (even in high dose) results in none to limited xGvHD.

 We also ascertained that a mouse with severe clinical cutaneous signs is involved with greater xGvHD-induced histopathologic changes in the skin, while a lethargic mouse is likely to be involved with damage to vital organs, especially the lungs and liver. For evaluation of xGvHD in animal models, two clinical and histopathological approaches can be used. Clinical assessment is non-invasive and easily applicable.^9,27^ It allows for rapid assessments and diagnosis, facilitating timely intervention.^28^ Nevertheless, xGvHD-induced clinical picture may be inconsistent due to the variable presentations and subjective assessments in grading the symptoms.^29^ Clinical signs may also overlap with other conditions, such as infections and ageing.^30^

 Histopathological evaluation provides more specific and confirmatory evidence of xGvHD,^31^ while presumably offering a gold standard. It allows for more objective staging of the disease and treatment planning in the clinical setting.^32^ Histopathological evidence often precedes clinical symptoms, especially in acute GvHD, providing a ‘lead time’ for preemptive treatment.^30^ On the other hand, the time required for biopsy and histological evaluation can delay the treatment in the clinical setting. It can also result in losing a number of study subjects unnecessarily in the preclinical setting. For histopathological evaluations, tissue specimens of the liver, intestine, skin, lung, and spleen have been commonly recommended.^33,34^ We found that liver and lung specimens provide more accurate and reproducible results in evaluating xGvHD of T cell-treated severely immunodeficient mice; and their results are more compatible with clinical presentations and immunohistochemical assays.

 The results of the present study may be undermined by some limitations. First of all, severely immunodeficient mice may not completely recapitulate the human immune system.^35^ Second, the onset of xGvHD can differ across different models and immunodeficient mouse strains.^36^ And in a broader context, different mouse strains, transplantation protocols, and tumor types can all introduce variability in the onset and severity of xGvHD, making it challenging to draw consistent conclusions.^37^

## Conclusion

 The occurrence of xGVHD is an unwanted complication following preclinical evaluation of human-derived T cell-based therapies in immunodeficient animal models. This may be averted by the use of CD4^+^T cell-depleted grafts. Moreover, optimal dose selection in preclinical evaluation of T cell-based therapies may minimize xGvHD severity and risks but this needs to be further evaluated. Implementing a standardized histopathological scoring system can offer a more objective assessment of the impacts of xGvHD on the models and differentiating GvH from GvT effects. Histopathological scoring of the lungs and liver provides a more precise and reliable assessment of xGvHD in immunodeficient strains undergoing human-derived T cell-based therapies.

## Supplementary Files

Supplementary file 1 contains Figure S1.
