# Supplementary material for: Clinicopathologic Effects of Xenogeneic GvHD Induced by Adoptively Transferred Human-Derived T Cells in Severely Immunodeficient Mice
Source: Arch Iran Med. 2024 Dec 1;27(12):683–92. doi: 10.34172/aim.28597 (PMC11786209; doi:10.34172/aim.28597)
Supplement: Supplementary file 1 — contains Figure S1. [file aim-27-683-s001.pdf]

Arch Iran Med. December 2024;27(12):683-S1

10.34172/aim.28597

http://journalaim.com

Supplementary file 1

Supplementary material of: Ashraf H, et al. Clinicopathologic Effects of Xenogeneic GvHD Induced by Adoptively Transferred Human-derived T Cells in Severely Immunodeficient Mice. Arch Iran Med. 2024.

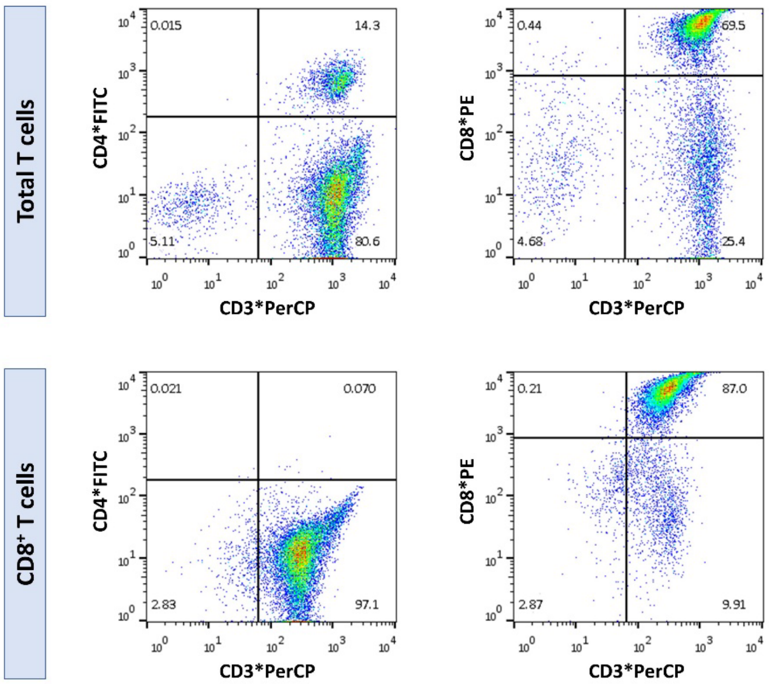

Supp. Fig. 1. Flow cytometry of total T cell and CD8+ T cell injectable products. The cellular products were immunophenotyped based on CD3, CD4, and CD8 surface antigens.

Figure S1. Flow cytometry of total T cell and CD8$^+$ T cell injectable products. The cellular products were immunophenotyped based on CD3, CD4, and CD8 surface antigens**.**
